# Supplementary material for: Phase separation of Nur77 mediates celastrol-induced mitophagy by promoting the liquidity of p62/SQSTM1 condensates
Source: Nat Commun. 2021 Oct 13;12:5989. doi: 10.1038/s41467-021-26295-8 (PMC8514450; doi:10.1038/s41467-021-26295-8)
Supplement: Supplementary file 1 — Supplementary Information [file 41467_2021_26295_MOESM1_ESM.pdf]

Supplementary Figure 1

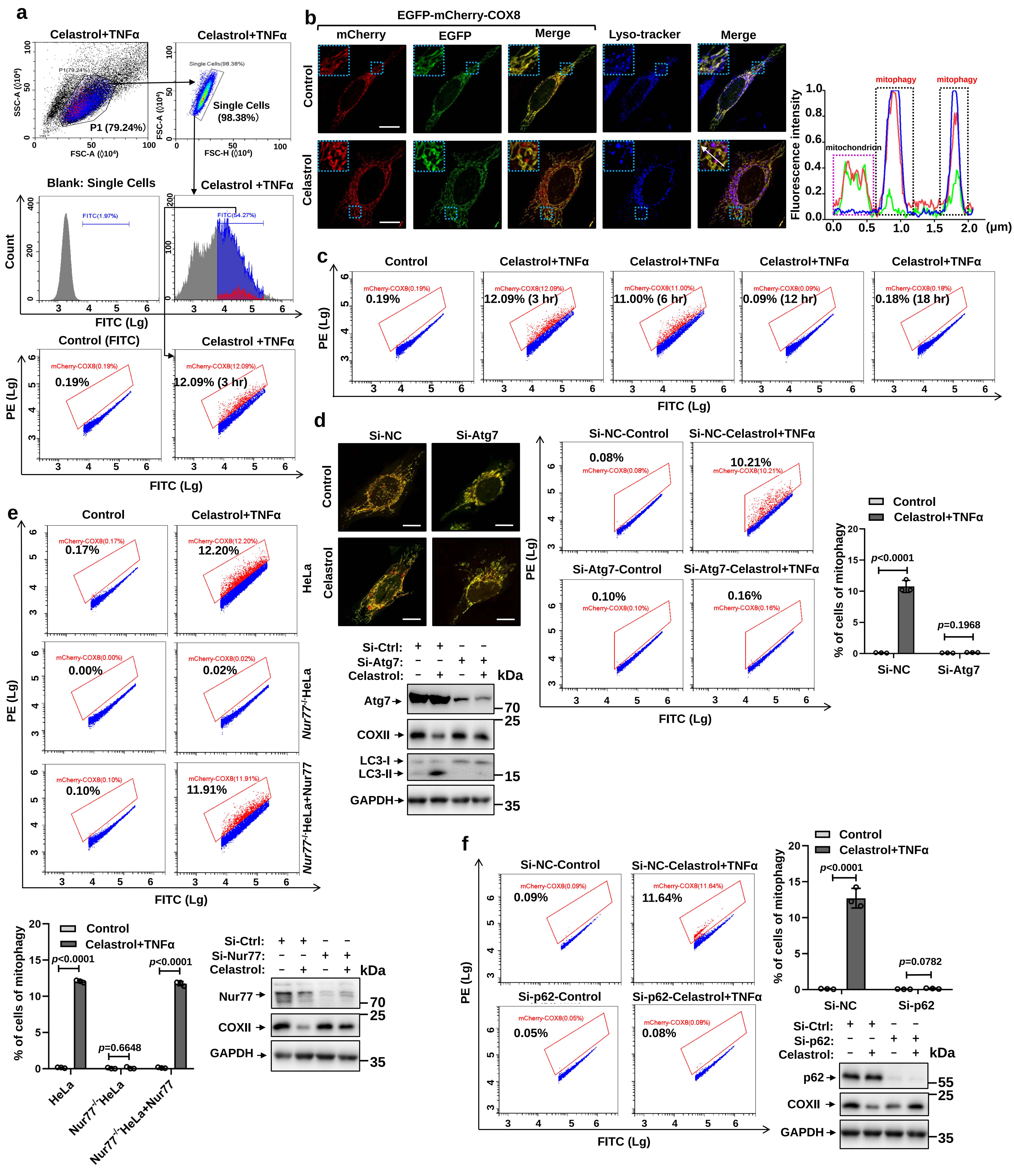

g

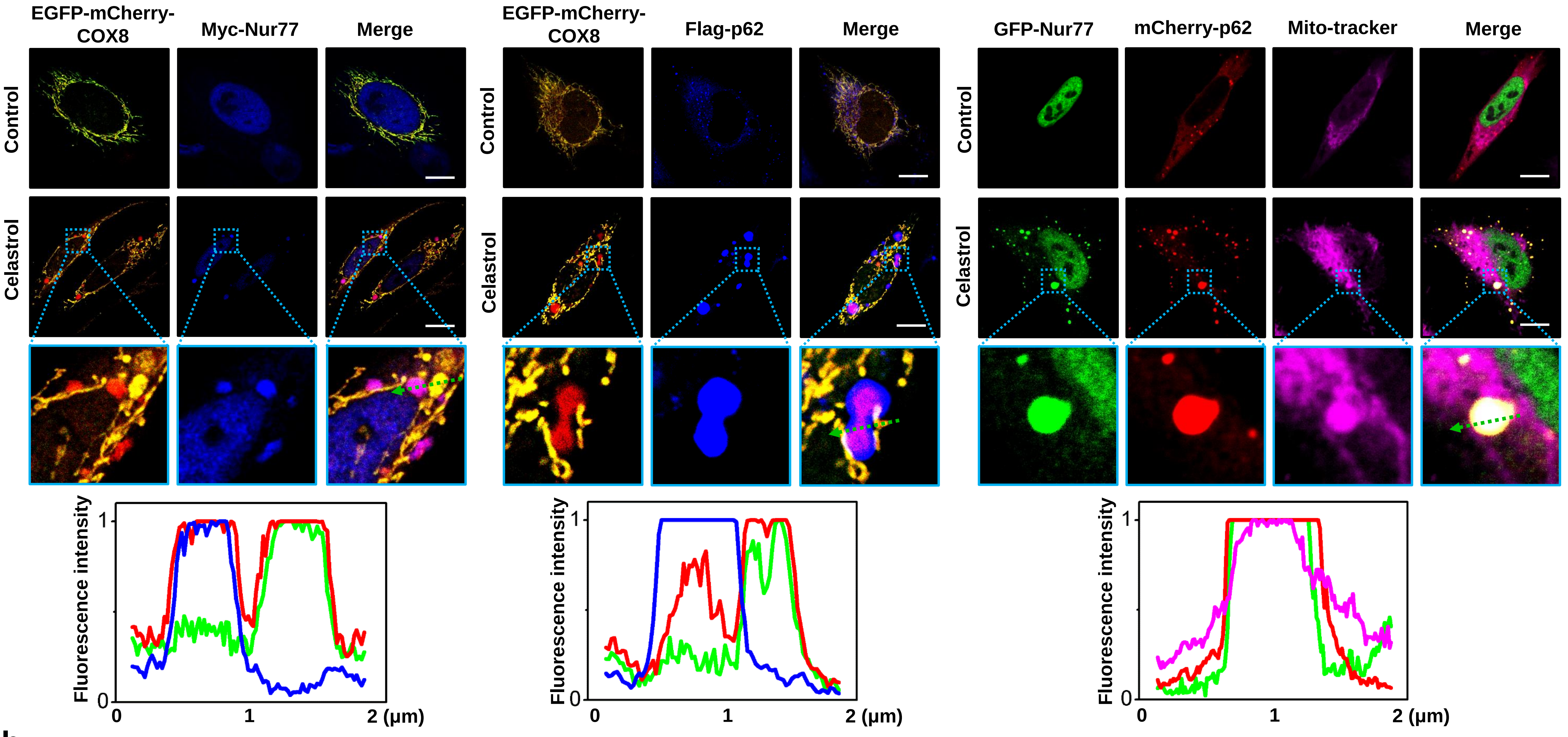

EGFP-mCherry-COX8
Myc-Nur77
Merge
Flag-p62
GFP-Nur77
mCherry-p62
Mito-tracker
Control
Celastrol
Fluorescence intensity
1
0
0
1
2 (μm)

h

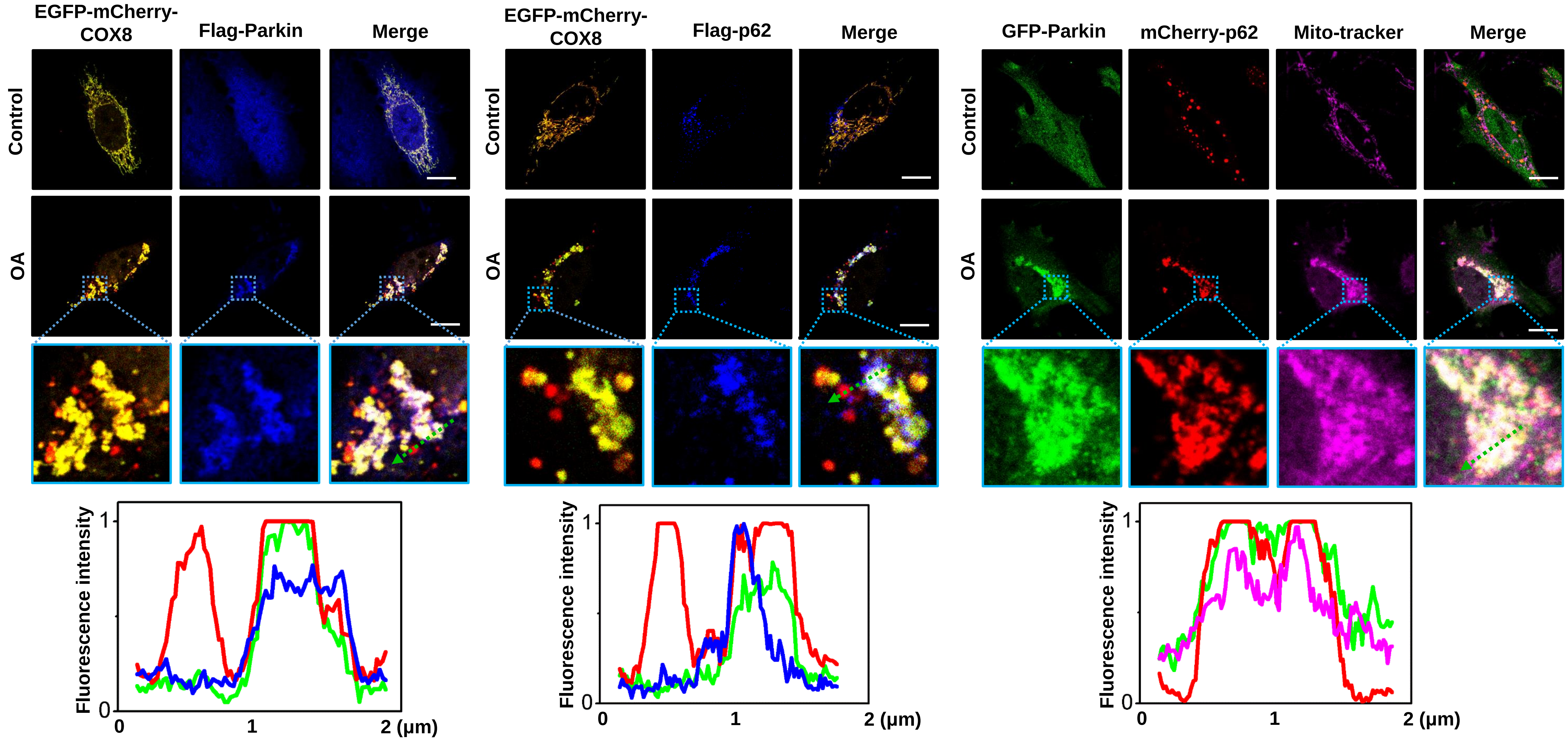

EGFP-mCherry-COX8
Flag-Parkin
Merge
Flag-p62
GFP-Parkin
mCherry-p62
Mito-tracker
Control
OA
Fluorescence intensity
1
0
0
1
2 (μm)

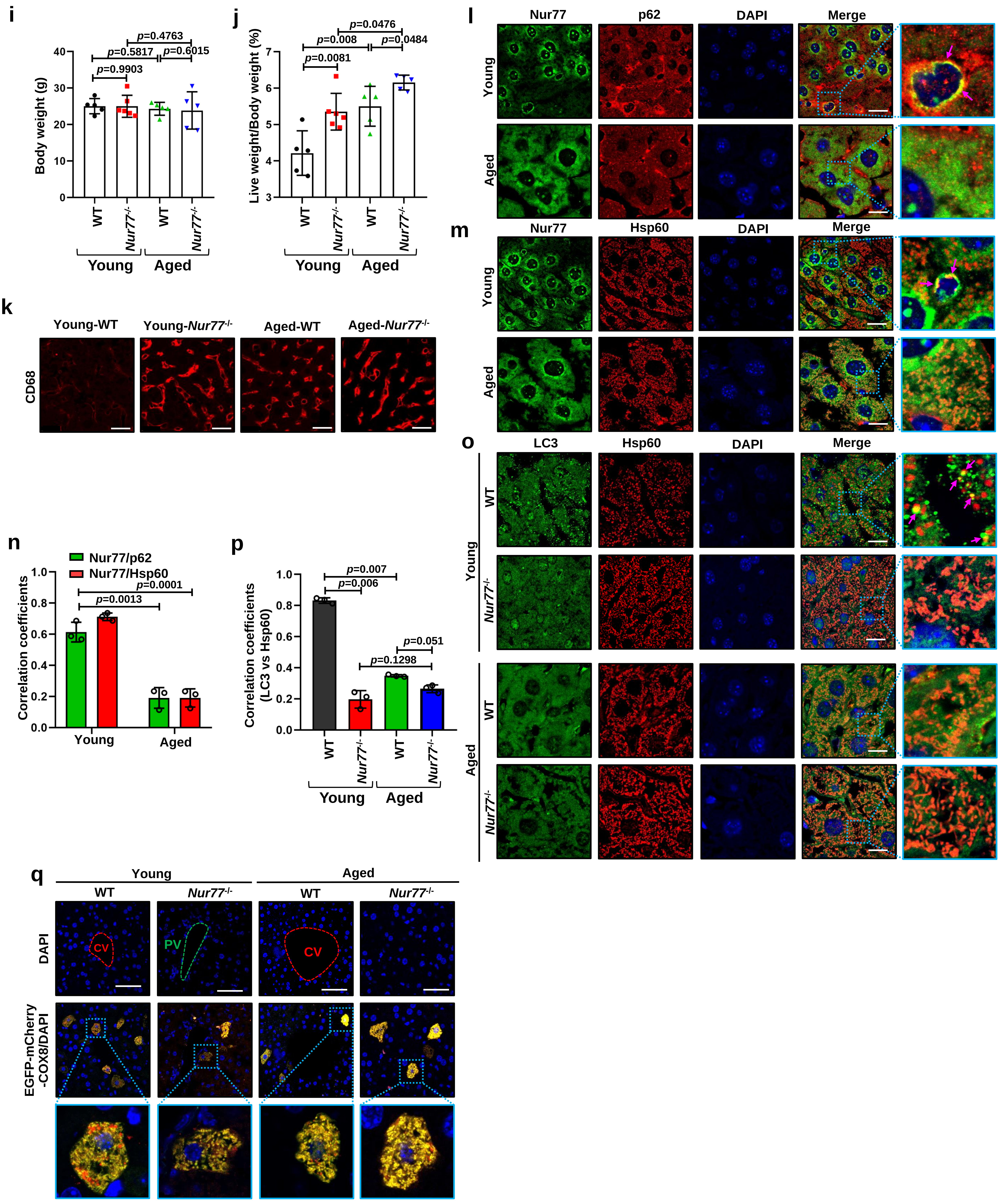
i
Body weight (g)
p=0.4763
p=0.5817
p=0.6015
p=0.9903
WT
Nur77-/-
Young
Aged
j
Live weight/Body weight (%)
p=0.0476
p=0.008
p=0.0484
p=0.0081
k
Young-WT
Young-Nur77-/-
Aged-WT
Aged-Nur77-/-
CD68
l
Nur77
p62
DAPI
Merge
m
Hsp60
n
Nur77/p62
Nur77/Hsp60
Correlation coefficients
p=0.0001
p=0.0013
p
Correlation coefficients (LC3 vs Hsp60)
p=0.007
p=0.006
p=0.051
p=0.1298
o
LC3
q
CV
PV
EGFP-mCherry -COX8/DAPI

Supplementary Figure 1. Characterization of Nur77/p62-mediated mitophagy.

a Gate setting of FACS plots of EGFP-mCherry-COX8 assay. EGFP-mCherry-COX8 positive cells were first gated (gate: P1) based on forward scatter area (FSC-A) and side scatter area (SSC-A), followed by doublet discrimination based on FSC height (FSC-H) by FSC-A (gate: Single Cells). EGFP-mCherry-COX8 positive cells were determined using untransfected controls (gate: FITC). Measurements EGFP-mCherry-COX8 were made using dual-excitation ratiometric pH measurements at FITC (488 nm, pH 7) and PE (561 nm, pH 4) lasers respectively. For each sample, 10,000 events of FITC positive cells were collected and subsequently gated with appropriate controls to detect mCherry-COX8 exposure cells but not EGFP-mCherry-COX8 double-positive cells.

b HeLa cells transfected with EGFP-mCherry-COX8 were treated with or without celastrol (2 μM) and TNFα (20 ng/ml) for 1 hr, and lysosomes were stained by lysosome tracker. Fluorescence analysis of EGFP-mCherry-COX8 by using 488 nm and 561 nm laser. When merged, normal mitochondria are yellow, whereas mitochondria engulfed into acidic lysosomes environment show red-only fluorescence. White arrow indicates the fluorescence intensity analysis showing that the red-only puncta were co-localized with lysosomes. Black dotted box shows mitophagy, and purple dotted box shows mitochondria. Scale bar, 10μm.

c Time-course analysis of the effect of celastrol in inducing mitophagy in HeLa cells transfected with EGFP-mCherry-COX8 were treated with or without celastrol and TNFα as indicated. Mitophagy was analyzed by flow cytometry.

d Representative images and quantification of celastrol-induced mitophagy in HeLa cells transfected with control (Si-NC) or Atg7 siRNA (Si-Atg7) and analyzed for celastrol induction of mitophagy by EGFP-mCherry-COX8 assay as described in Methods. Left lower panel shows the expression of Atg7, LC3-II and mitochondrial membrane COXII proteins in HeLa cells transfected with control and Atg7 siRNA and treated with or without celastrol and TNFα for 12 hr. Two-tailed unpaired Student's t test was used for statistical analysis, and data are presented as mean values ± SEM (n=3 independent experiments). Scale bar, 10 μm.

e Quantification of celastrol-induced mitophagy in HeLa, *Nur77*$^{-/-}$HeLa, and *Nur77*$^{-/-}$HeLa cells transfected with Myc-Nur77 by EGFP-mCherry-COX8 assay. Lower panel shows celastrol-induced degradation of mitochondrial membrane proteins depends on the expression of Nur77. HeLa cells transfected with or without the indicated siRNA were treated with or without celastrol and TNFα for 12 hr, and analyzed by Western blot. Two-tailed unpaired Student's t test was used for statistical analysis, and data are presented as mean values ± SEM (n=3 independent experiments).

f Quantification of celastrol-induced mitophagy in HeLa cells transfected with control or p62 siRNA by flow cytometry. Right lower panel shows Celastrol-induced degradation of COXII depends on the expression of p62. HeLa cells transfected with or without the indicated siRNA were treated with or without celastrol and TNFα for 12 hr, and analyzed by Western blot. Two-tailed unpaired Student's t test was used for statistical analysis, and data are presented as mean values ± SEM (n=3 independent experiments).

g HeLa cells transfected with the indicated expression vectors were treated with or without celastrol and TNFα for 6 hr, and examined by immunostaining. Representative images showing celastrol-induced colocalization of transfected Myc-Nur77 (left panel) or Flag-p62 (middle panel) with red puncta of EGFP-mCherry-COX8 and colocalization of transfected GFP-Nur77 and mCherry-p62 with mitochondria (right panel). Arrows (green) indicate the plane used for examining the colocalization. Scale bar, 10 μm.

h HeLa cells transfected with the indicated expression vectors were treated with or without oligomycin (10 μM) and antimycin A (4 μM) (OA) for 6 hr, and examined by immunostaining. Representative images showing lack of colocalization of transfected Flag-Parkin (left panel) or Flag-p62 (middle panel) with red puncta of EGFP-mCherry-COX8, and the colocalization of transfected GFP-Parkin and mCherry-p62 with mitochondria (right panel) in cells undergoing OA-induced mitophagy. Line profiles of fluorescence intensities (green) indicates lack of colocalization of Flag-Parkin and Flag-p62 with red puncta of EGFP-mCherry-COX8. Scale bar, 10 μm.

i Body weight of wild-type and *Nur77*$^{-/-}$mice in aging model. Two-tailed unpaired Student's t test was used for statistical analysis, and data are presented as mean values ± SEM (n=5 mice per group).

j Ratio of liver weight and body weight from wild-type and *Nur77*$^{-/-}$mice in aging model. Two-tailed unpaired Student's t test was used for statistical analysis, and data are presented as mean values ± SEM (n=5 mice per group).

k Immunostaining of inflammatory cytokine CD68 in wild-type and *Nur77*$^{-/-}$mice in aging model. Scale bar, 25 μm.

l-n Representative images show colocalization of Nur77 with p62 and Hsp60 in young and aged mice. Pearson's correlation coefficients of Nur77 with p62 and Hsp60 were also shown. Purple arrows indicate the colocalization of Nur77 with p62 and Hsp60. Two-tailed unpaired Student's t test was used for statistical analysis, and data are presented as mean values ± SEM (n=3 biologically independent samples). Scale bar, 10 μm.

o-p. Representative images illustrate colocalization of LC3 with Hsp60 in the livers from wild-type or *Nur77*$^{-/-}$mice in aging model. Purple arrows indicate the colocalization of LC3 with mitochondria. Two-tailed unpaired Student's t test was used for statistical analysis, and data are presented as mean values ± SEM (n=3 biologically independent samples). Scale bar, 10 μm.

q. Representative images of EGFP-mCherry-COX8 shows mitophagy in the liver from aging model. The red dotted line indicates central vein (CV). The green dotted line indicates portal vein (PV). Scale bar, 30 μm.

Supplementary Figure 2

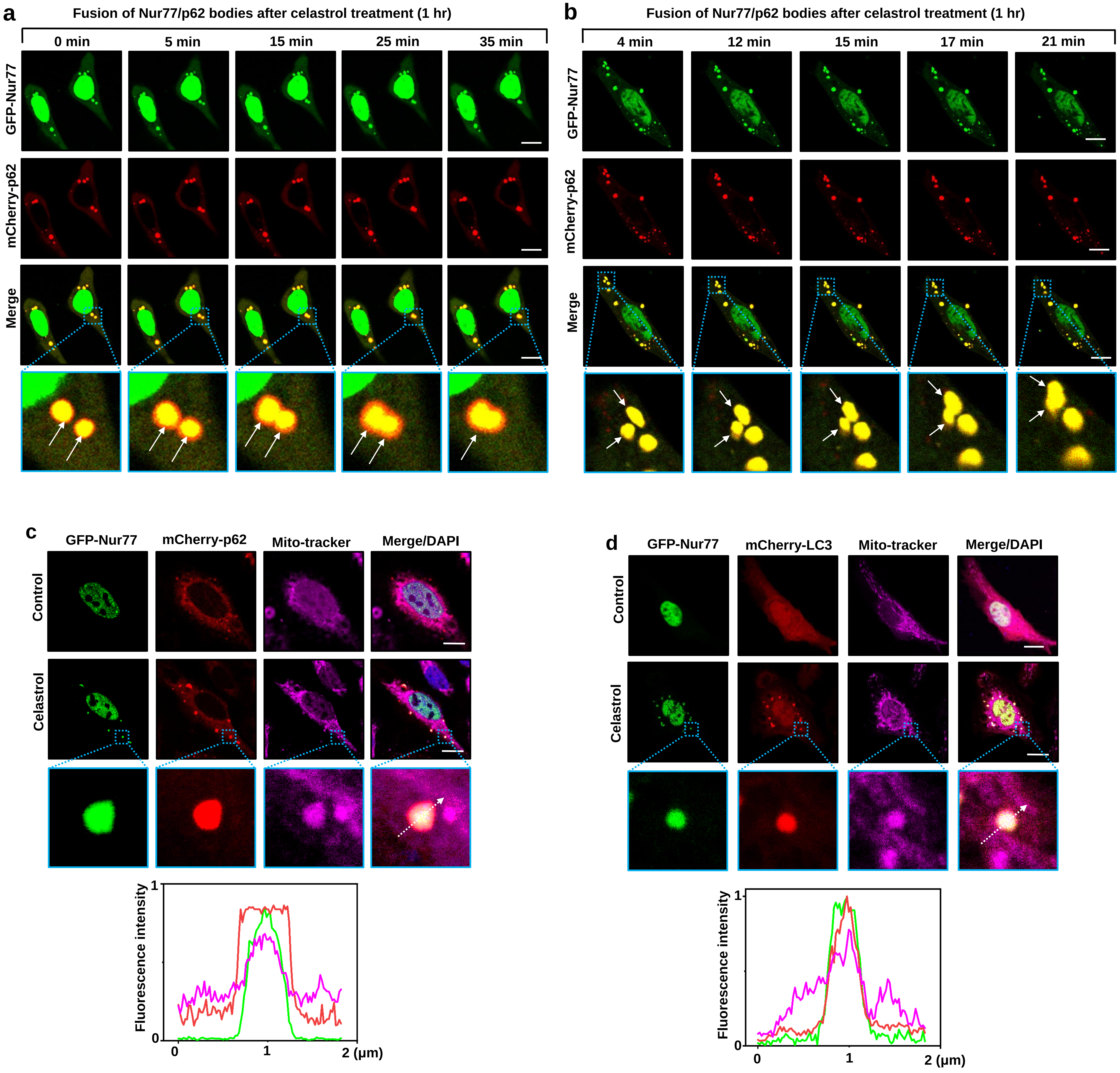

Supplementary Figure 2. Characterization of Nur77/p62 condensates.

a-b Stills of movies showing fusion of GFP-Nur77 and mCherry-p62 droplets in HeLa cells after treatment with celastrol (2 μM) and TNFα (20 ng/ml) for 1 hr as indicated by the arrows. (see also Supplementary Movies 2 and 3). Scale bar, 10 μm.
c Celastrol-induced Nur77/p62 condensates contain mitochondria. HeLa cells transfected with GFP-Nur77 and mCherry-p62 were treated with or without celastrol (2 μM) and TNFα (20 ng/ml) for 1 hr, and examined by confocal microscopy. Representative images show colocalization of GFP-Nur77 with mCherry-p62 and mitochondria in HeLa cells undergoing celastrol-induced mitophagy. Enlarged view of inset is also shown. The fluorescence intensity was analyzed. Scale bar, 10 μm.
d Celastrol-induced Nur77 condensates colocalize with mitochondria and LC3. HeLa cells transfected with GFP-Nur77 and mCherry-LC3 were treated with or without celastrol (2 μM) and TNFα (20 ng/ml) for 1 hr, and examined by confocal microscopy. Representative images show colocalization of GFP-Nur77 with mCherry-LC3 and mitochondria in HeLa cells undergoing celastrol-induced mitophagy. Enlarged view of inset is also shown. The fluorescence intensity was analyzed. Scale bar, 10 μm.

Supplementary Figure 3

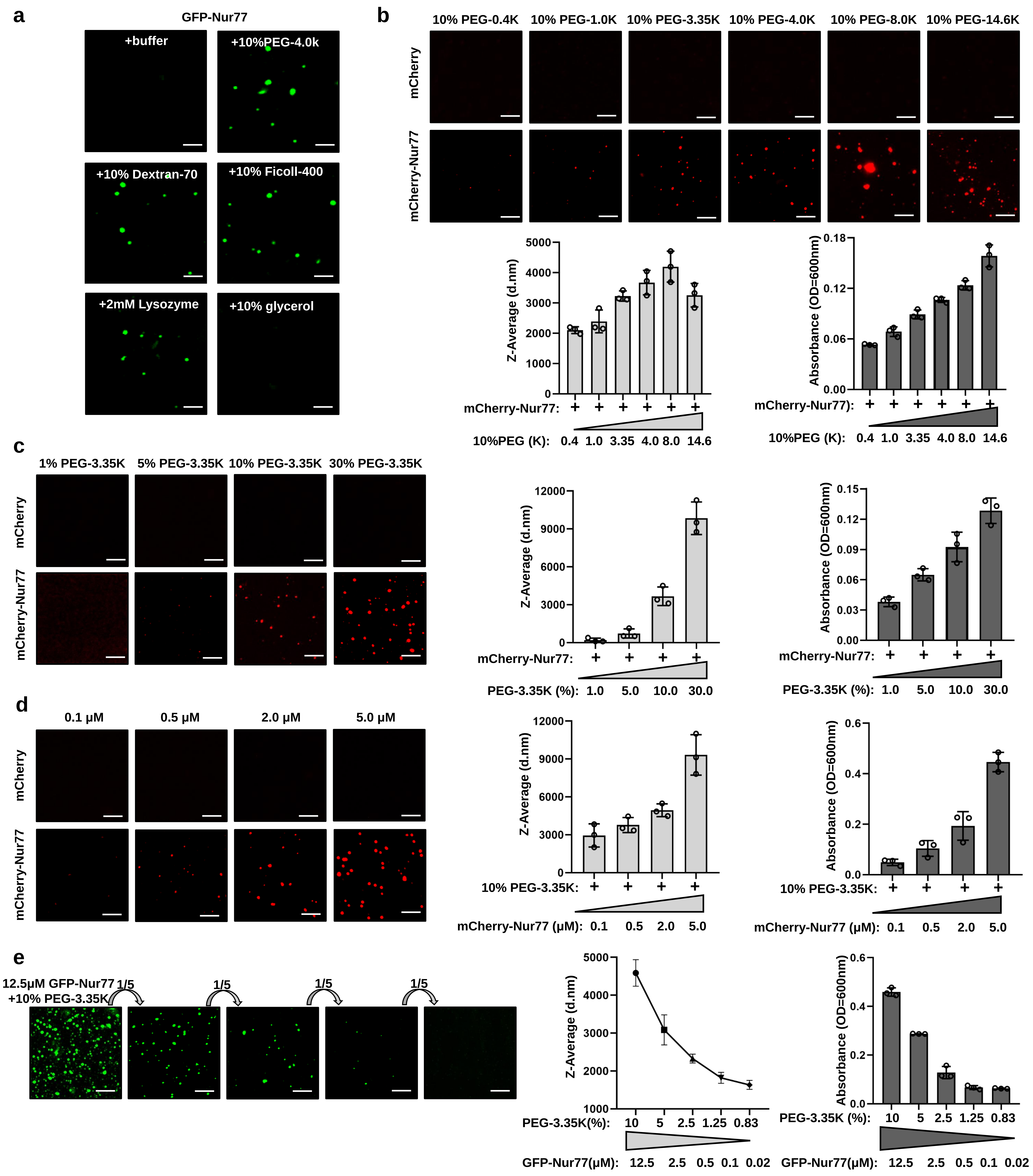

Supplementary Figure 3. Characterization of Nur77 condensates.

a The formation of Nur77 droplet in different crowding agents. Purified GFP-Nur77 protein (1 μM) was incubated with the indicated crowding agents for 2 min then imaged. Scale bar, 5μm.

b Nur77 droplet formation is dependent on the molecular weight of crowing agent. Purified mCherry-Nur77 (0.5 μM) was incubated in 10% PEG solution with different molecular weight (0.4–14.6 kDa) for 10 min, then imaged. Upper panels: Representative images of droplet formation of mCherry-Nur77 in PEG solution with different molecular weight. Bottom panel: The size and turbidity of mCherry-Nur77 droplets was calculated and then normalized. Data are presented as mean values ± SEM (n=3 independent experiments). Scale bar, 5 μm.

c Effect of PEG concentration on Nur77 droplet formation. Concentration dependent effect of PEG-3.35K on mCherry-Nur77 droplet formation. Left: Representative images of mCherry-Nur77 droplet at the indicated PEG-3.35K concentration. Right: The size and turbidity of droplets was calculated and normalized. Data are presented as mean values ± SEM (n=3 independent experiments). Scale bar, 5 μm.

d Nur77 droplet formation is dependent on Nur77 concentration. Concentration dependent effect of Nur77 protein on droplet formation. Left: Representative images of mCherry-Nur77 droplets at the indicated mCherry-Nur77 concentration. Right: The size and turbidity of mCherry-Nur77 droplets was calculated and normalized. Data are presented as mean values ± SEM (n=3 independent experiments). Scale bar, 5μm.

e Nur77 droplet formation is reversible. GFP-Nur77 (12.5 μM) droplets were formed in 10% PEG-3.35K for 10 min, followed by a 1:5 dilution (diluted 1/5) in buffer. Left: Representative images of GFP-Nur77 droplets at the indicated dilution of GFP-Nur77 protein concentration. Middle: The size of GFP-Nur77 droplets were calculated. Right: The turbidity of GFP-Nur77 droplets were calculated and normalized. Data are presented as mean values ± SEM (n=3 independent experiments). Scale bar, 5 μm.

Supplementary Figure 4

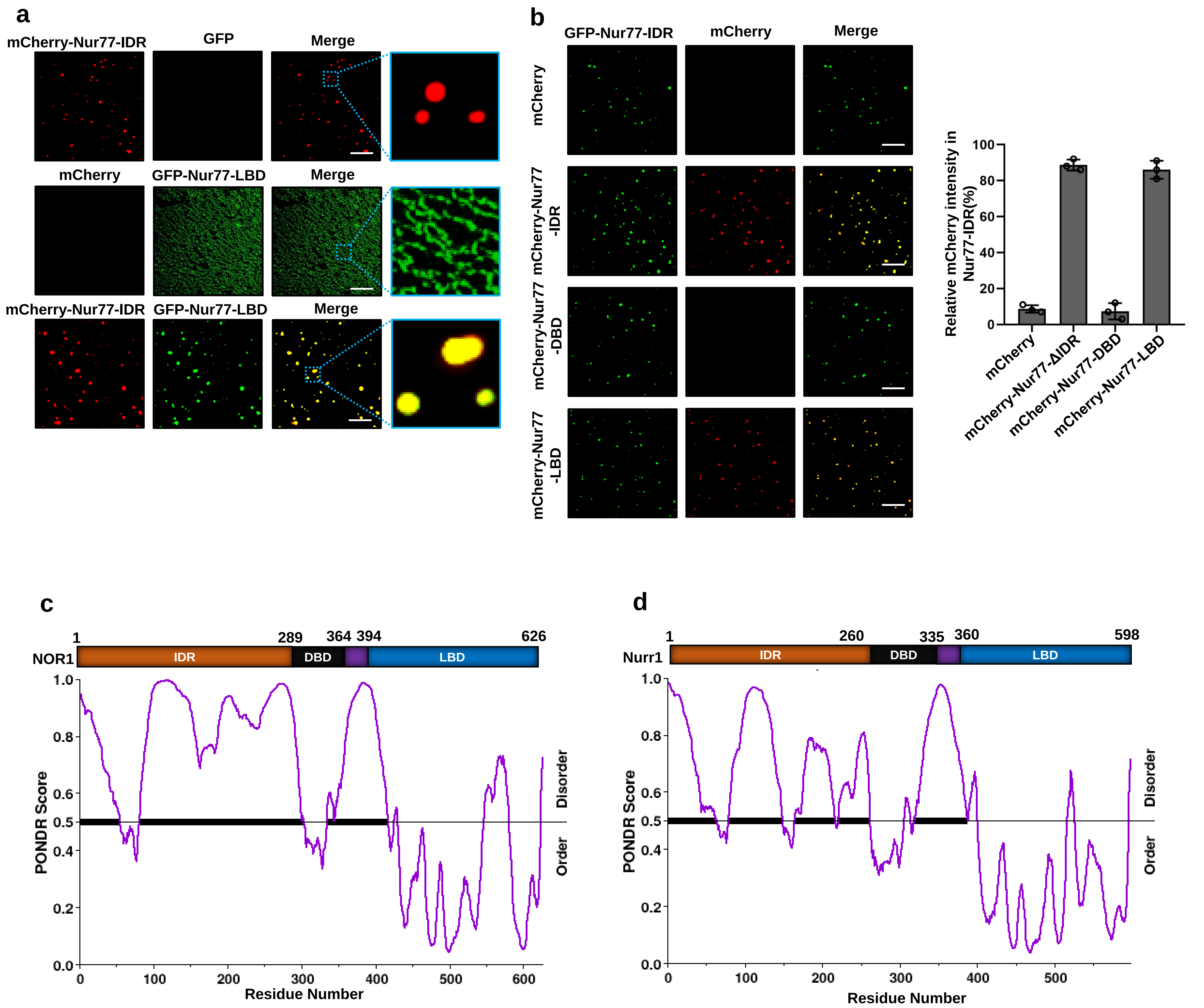

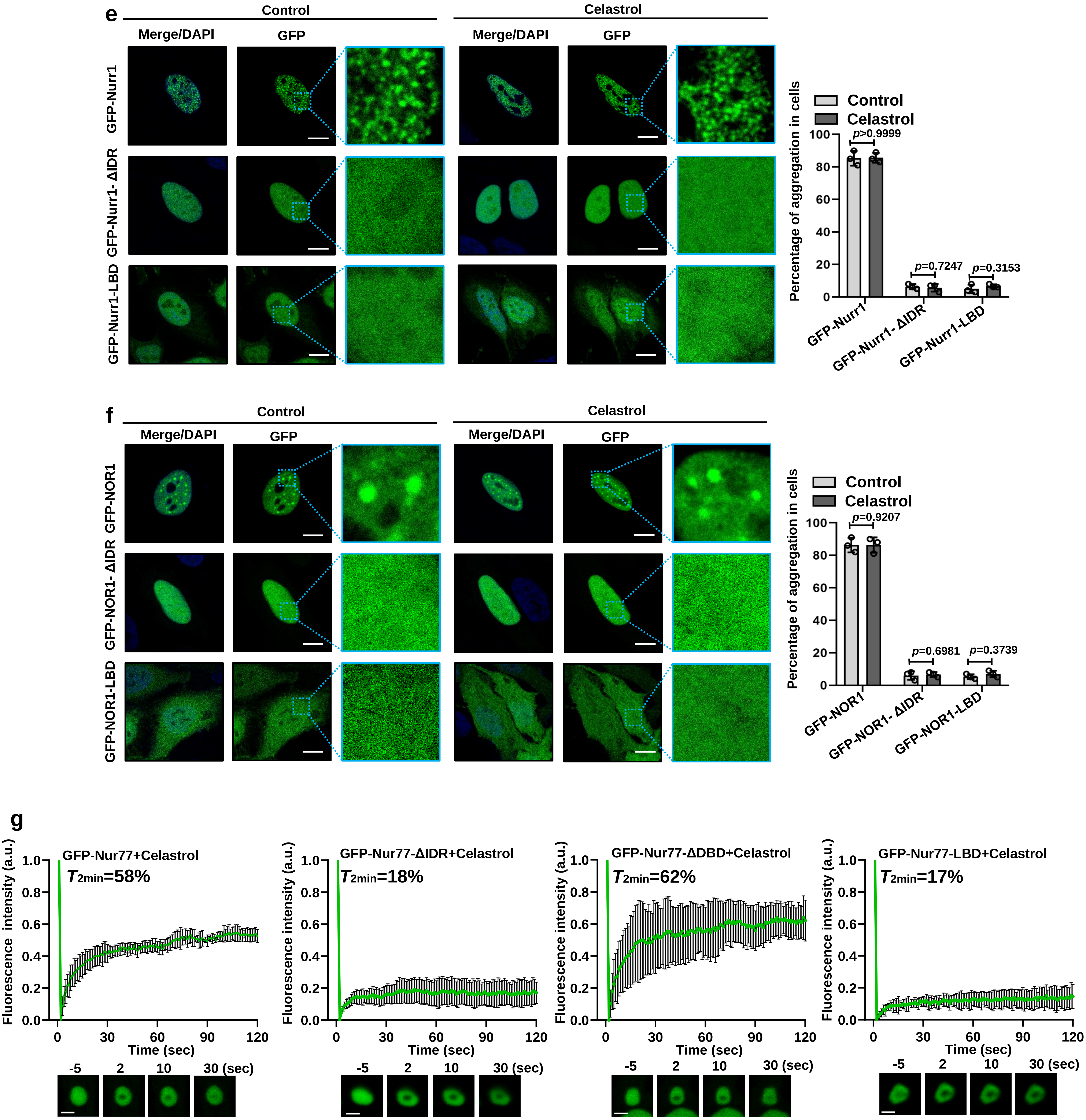

e
Control
Celastrol
Merge/DAPI
GFP
GFP-Nurr1
GFP-Nurr1- ΔIDR
GFP-Nurr1-LBD
Percentage of aggregation in cells
p>0.9999
p=0.7247
p=0.3153
f
GFP-NOR1
GFP-NOR1- ΔIDR
GFP-NOR1-LBD
p=0.9207
p=0.6981
p=0.3739
g
GFP-Nur77+Celastrol
T2min=58%
GFP-Nur77-ΔIDR+Celastrol
T2min=18%
GFP-Nur77-ΔDBD+Celastrol
T2min=62%
GFP-Nur77-LBD+Celastrol
T2min=17%
Fluorescence intensity (a.u.)
Time (sec)
-5
2
10
30 (sec)

Supplementary Figure 4. IDR of Nur77 family members is important for their phase separation.

a-b Representative images and quantification of Nur77-IDR in modulating phase separation of Nur77-DBD and Nur77-LBD in vitro. Purified GFP-Nur77-IDR (2 μM) was incubated with mCherry, mCherry-Nur77-IDR, mCherry-Nur77-DBD and mCherry-Nur77-LBD (2 μM) for 10 min in 10% PEG-3.35K, then imaged. Data are presented as mean values ± SEM (n=3 independent experiments). Scale bar, 5 μm.
c-d Intrinsic disorder tendency of NOR1 and Nurr1. IDR, intrinsic disorder region; DBD, DNA-binding domain; LBD, ligand-binding domain.
e-f Droplet formation of GFP-Nurr1 and GFP-NOR1 or mutants in HeLa cells treated with or without celastrol (2 μM). Right: quantification of diffusion or aggregation of GFP-Nurr1, GFP-NOR1, and their mutants. Two-tailed unpaired Student's t test was used for statistical analysis, and data are presented as mean values ± SEM (n=3 independent experiments). Scale bar, 10 μm.
g Representative images and quantification of fluorescence intensity recovery of GFP-Nur77 and mutant bodies. Data are presented as mean values ± SEM (n=3 independent experiments). Scale bar, 1.5 μm.

Supplementary Figure 5.

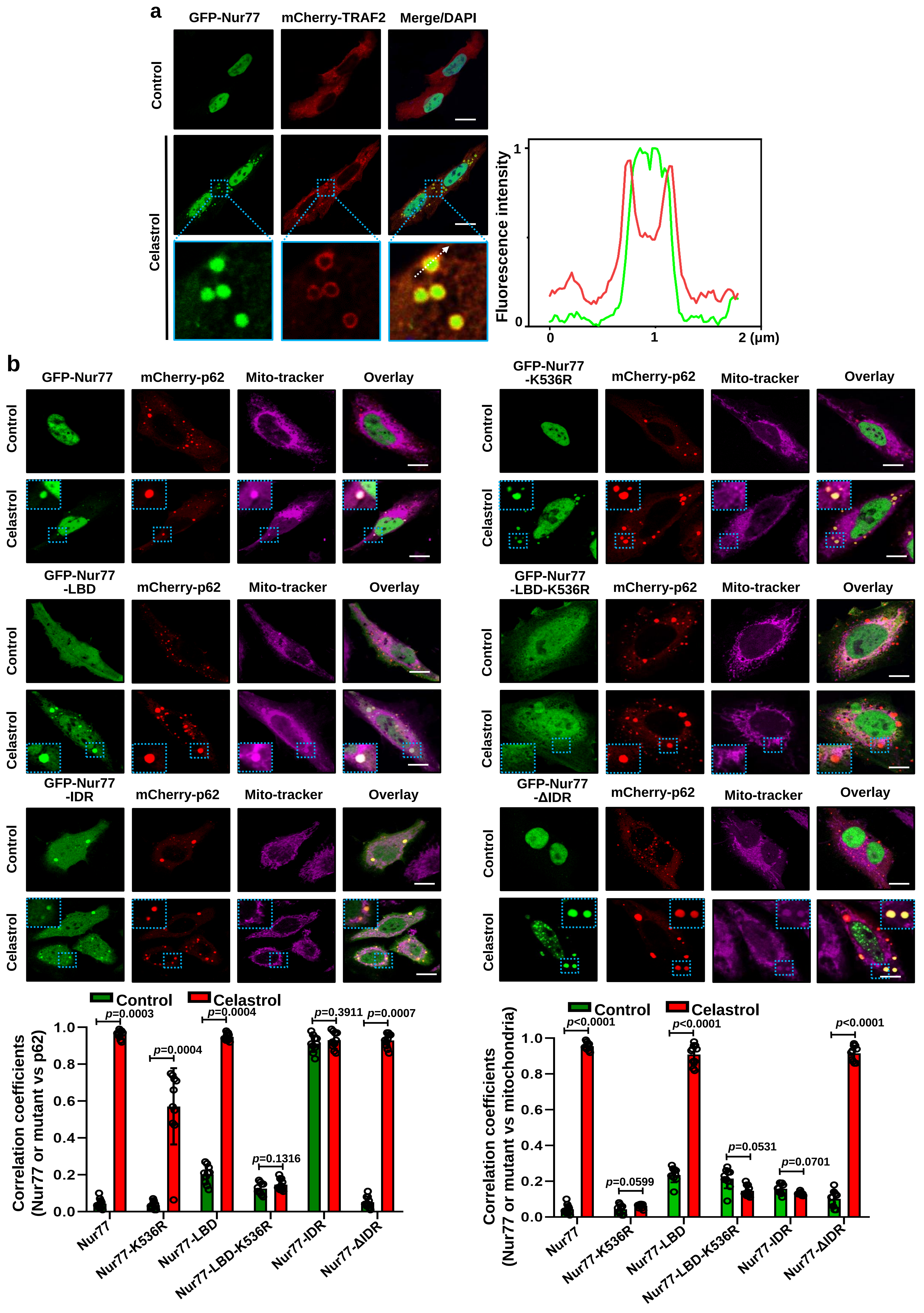

Supplementary Figure 5. Nur77 ubiquitination is required for celastrol-induced p62 localization at mitochondria.

a TRAF2 is recruited by Nur77 condensates. HeLa cells transfected with GFP-Nur77 and mCherry-TRAF2 were treated with or without celastrol (2 μM) and TNFα (20 ng/ml) for 1 hr. Representative images of GFP-Nur77 and mCherry-TRAF2 were revealed by confocal microscopy. Right, line scans of the indicated droplet containing GFP-Nur77 and mCherry-TRAF2 with mCherry-TRAF2 at the surface of GFP-Nur77 condensates. Scale bar, 10 μm.

b Nur77 ubiquitination-mediated p62 phase separation is required for sequestering mitochondria. HeLa cells transfected with mCherry-p62 and GFP-Nur77 or mutant were treated with or without celastrol (2 μM) and TNFα (20 ng/ml) for 1 hr and their subcellular localization was examined by confocal microscopy. Pearson's correlation coefficients of p62 with Nur77 or mutant at mitochondria were also shown. Two-tailed unpaired Student's t test was used for statistical analysis, and data are presented as mean values ± SEM (n=10 biologically independent samples). Scale bar, 10 μm.

Supplementary Figure 6

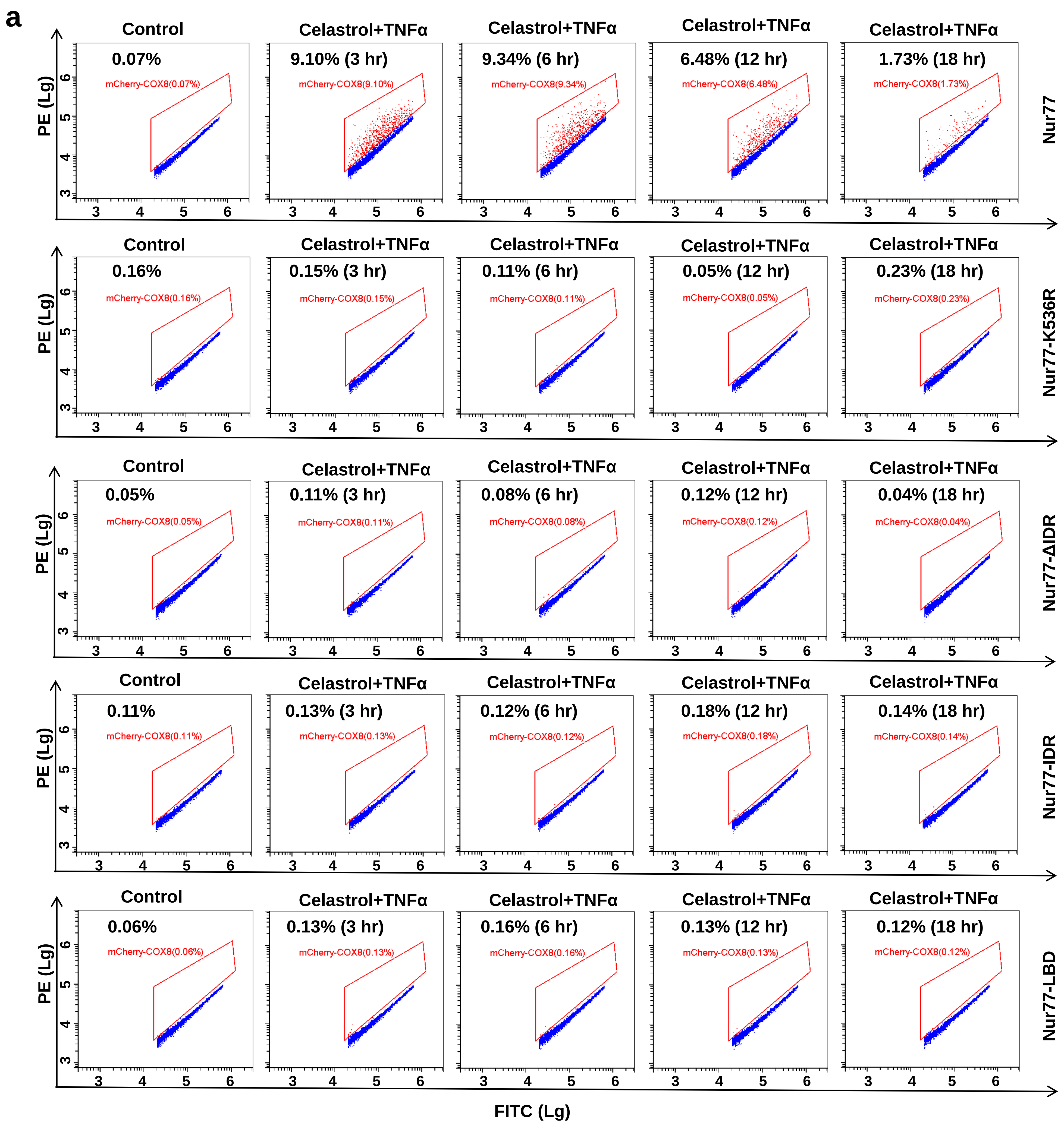

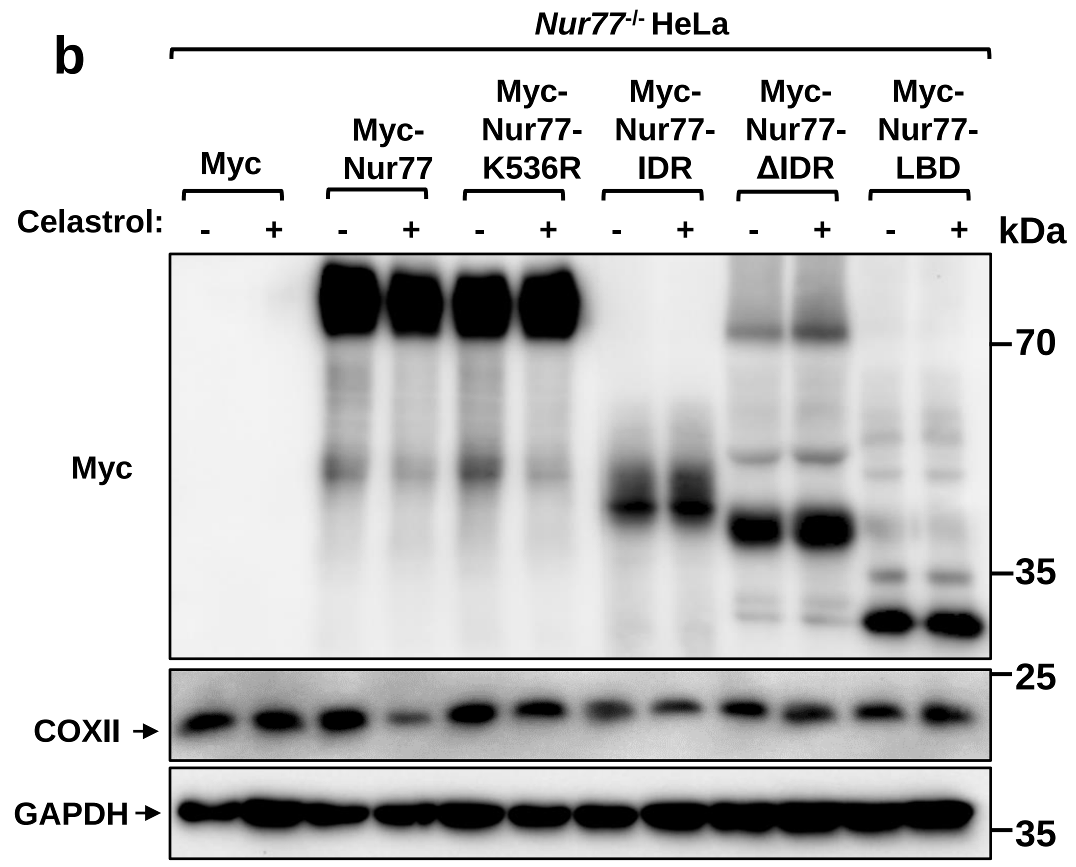

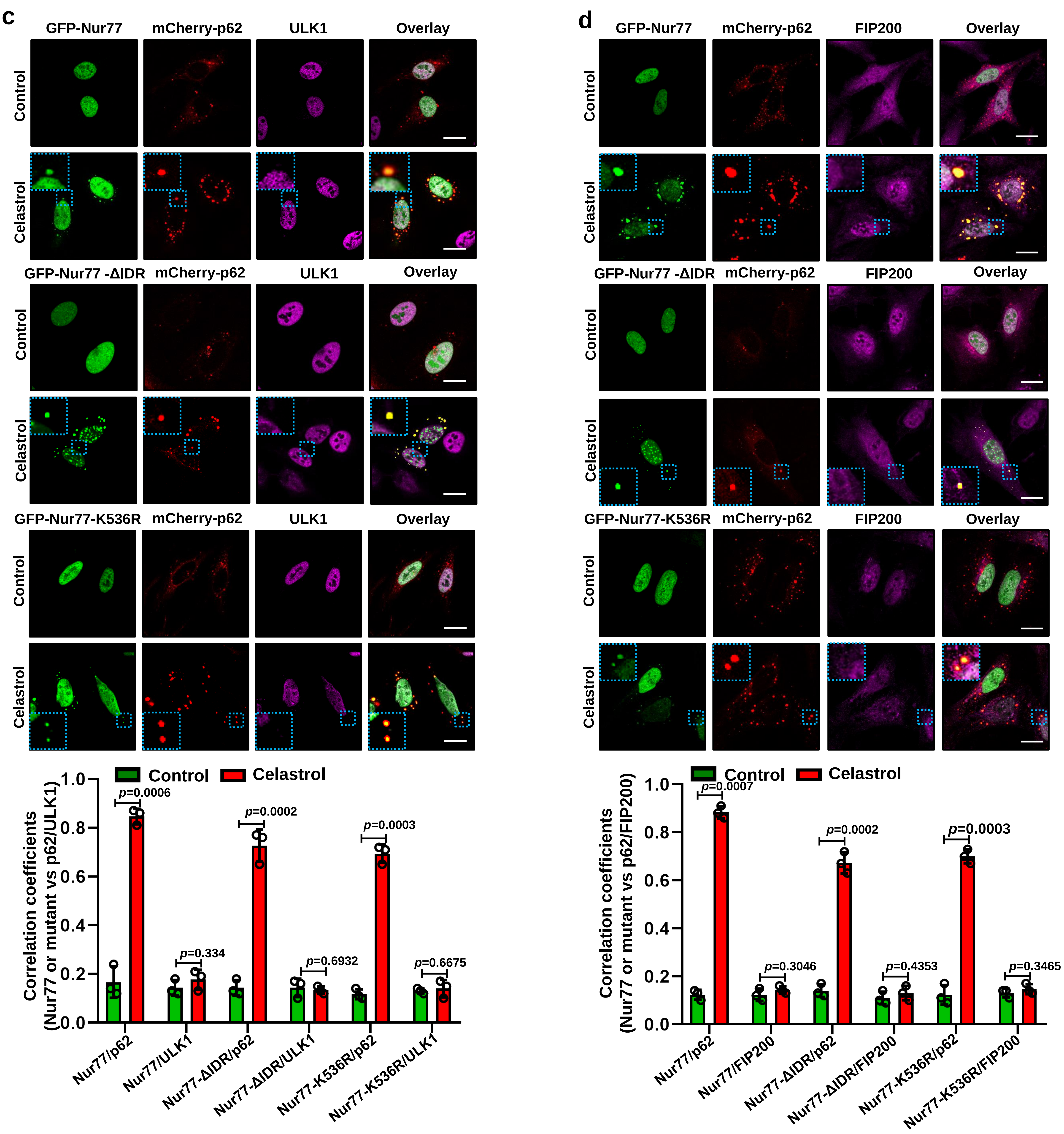
c
GFP-Nur77
mCherry-p62
ULK1
Overlay
Control
Celastrol
GFP-Nur77 -ΔIDR
mCherry-p62
ULK1
Overlay
Control
Celastrol
GFP-Nur77-K536R
mCherry-p62
ULK1
Overlay
Control
Celastrol
Control
Celastrol
Correlation coefficients
(Nur77 or mutant vs p62/ULK1)
1.0
0.8
0.6
0.4
0.2
0.0
p=0.0006
p=0.334
p=0.0002
p=0.6932
p=0.0003
p=0.6675
Nur77/p62
Nur77/ULK1
Nur77-ΔIDR/p62
Nur77-ΔIDR/ULK1
Nur77-K536R/p62
Nur77-K536R/ULK1
d
GFP-Nur77
mCherry-p62
FIP200
Overlay
Control
Celastrol
GFP-Nur77 -ΔIDR
mCherry-p62
FIP200
Overlay
Control
Celastrol
GFP-Nur77-K536R
mCherry-p62
FIP200
Overlay
Control
Celastrol
Control
Celastrol
Correlation coefficients
(Nur77 or mutant vs p62/FIP200)
1.0
0.8
0.6
0.4
0.2
0.0
p=0.0007
p=0.3046
p=0.0002
p=0.4353
p=0.0003
p=0.3465
Nur77/p62
Nur77/FIP200
Nur77-ΔIDR/p62
Nur77-ΔIDR/FIP200
Nur77-K536R/p62
Nur77-K536R/FIP200

e

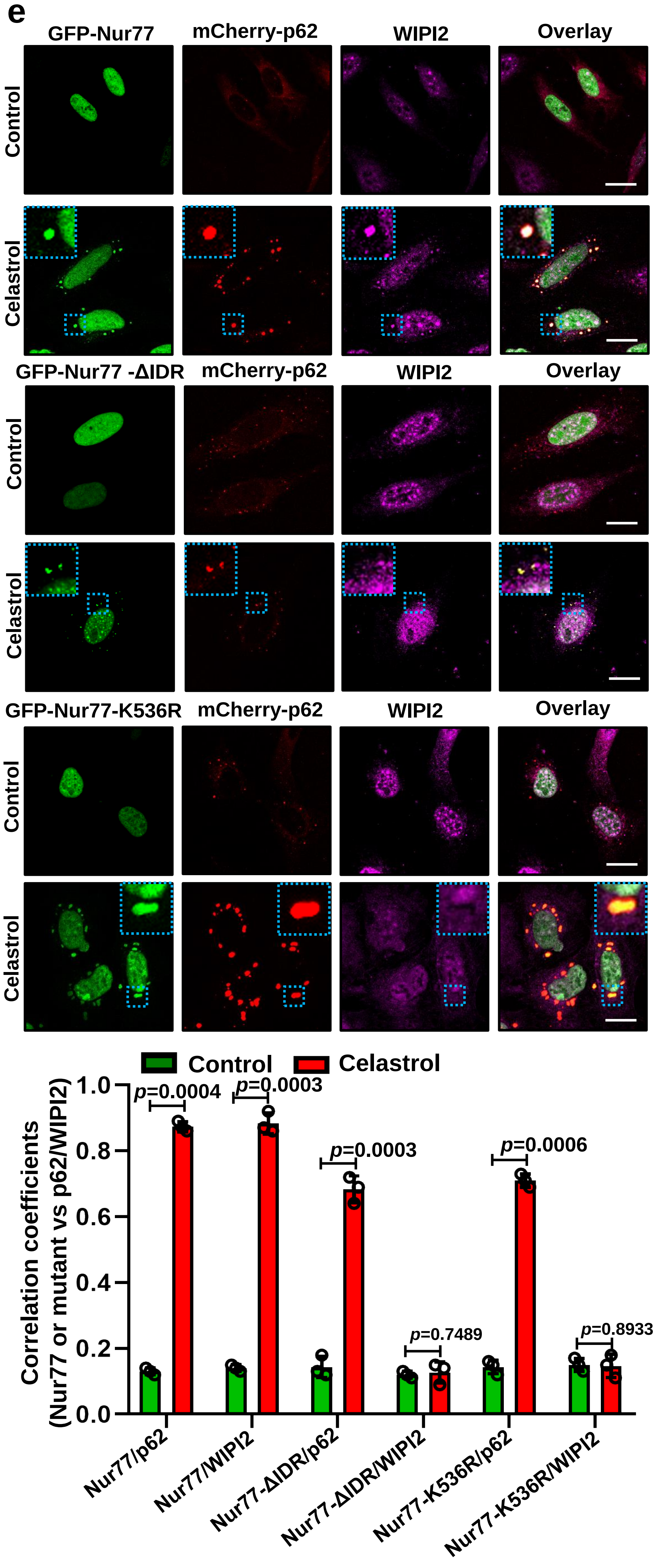
GFP-Nur77
mCherry-p62
WIPI2
Overlay
Control
Celastrol
GFP-Nur77 -ΔIDR
mCherry-p62
WIPI2
Overlay
Control
Celastrol
GFP-Nur77-K536R
mCherry-p62
WIPI2
Overlay
Control
Celastrol
Control
Celastrol
Correlation coefficients
(Nur77 or mutant vs p62/WIPI2)
1.0
0.8
0.6
0.4
0.2
0.0
p=0.0004
p=0.0003
p=0.0003
p=0.7489
p=0.0006
p=0.8933
Nur77/p62
Nur77/WIPI2
Nur77-ΔIDR/p62
Nur77-ΔIDR/WIPI2
Nur77-K536R/p62
Nur77-K536R/WIPI2

f

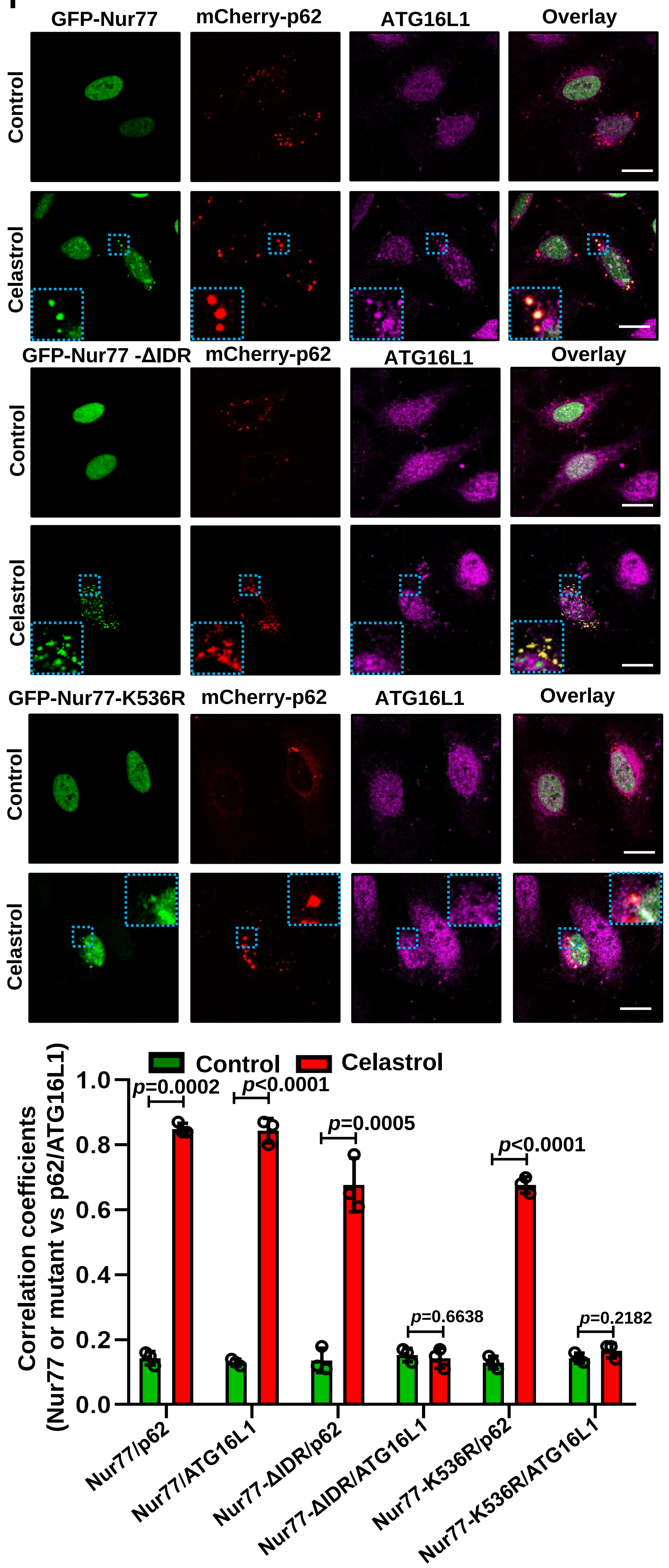
GFP-Nur77
mCherry-p62
ATG16L1
Overlay
Control
Celastrol
GFP-Nur77 -ΔIDR
mCherry-p62
ATG16L1
Overlay
Control
Celastrol
GFP-Nur77-K536R
mCherry-p62
ATG16L1
Overlay
Control
Celastrol
Control
Celastrol
Correlation coefficients
(Nur77 or mutant vs p62/ATG16L1)
1.0
0.8
0.6
0.4
0.2
0.0
p=0.0002
p<0.0001
p=0.0005
p=0.6638
p<0.0001
p=0.2182
Nur77/p62
Nur77/ATG16L1
Nur77-ΔIDR/p62
Nur77-ΔIDR/ATG16L1
Nur77-K536R/p62
Nur77-K536R/ATG16L1

Supplementary Figure 6. Nur77 ubiquitination-mediated p62 phase separation is not sufficient to complete celastrol-induced mitophagy.

a Structural integrity of Nur77 is required for celastrol-induced mitophagy. Time-course analysis of the effect of Nur77 or mutants in mediating celastrol-induced mitophagy. *Nur77*$^{-/-}$HeLa cells transfected with EGFP-mCherry-COX8 and the indicated Myc-Nur77 or mutant were treated with or without celastrol (2 μM) and TNFα (20 ng/ml) as indicated, and examined for mitophagy. The mitophagy was quantified using the EGFP-mCherry-COX8 marker and analyzed by flow cytometry.

b Characterization of the mitophagic effect of Nur77 mutants. HeLa cells depleted with Nur77 (*Nur77*$^{-/-}$ HeLa) were transfected with Nur77 and mutants (Nur77-K536R, Nur77-IDR, Nur77-ΔIDR, and Nur77-LBD), treated with or without celastrol (2 μM) and TNFα (20 ng/ml) for 12 hr, and analyzed by Western blot.

c-f Representative images showing colocalization of the indicated ATG proteins with p62, Nur77, or mutants in the presence or absence of celastrol. Pearson's correlation coefficients of Nur77 or mutants with p62 and ULK1, FIP200, WIPI2 and ATG16L1 were also shown. Two-tailed unpaired Student's t test was used for statistical analysis, and data are presented as mean values ± SEM (n=3 independent experiments). Scale bar, 10 μm.

Supplementary Figure 7

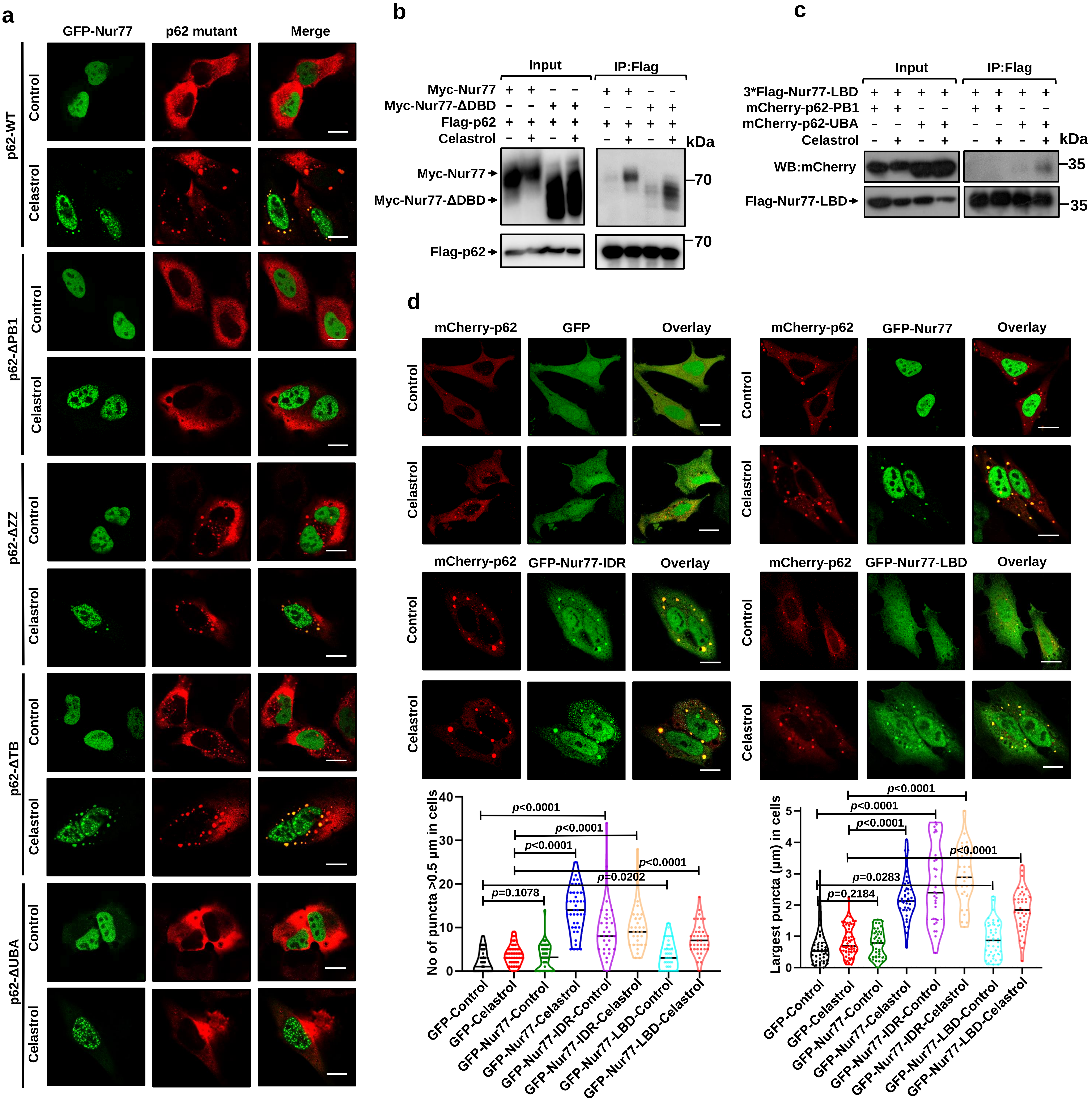

Supplementary Figure 7. Interaction between IDR of Nur77 and PB1 of p62 promotes the expansion and liquidity of Nur77/p62 condensates*.*

a PB1 and UBA of p62 are required for p62 colocalization with Nur77. Colocalization of GFP-Nur77 with Flag-p62 or mutants was analyzed in HeLa cells treated with or without celastrol (2 μM) for 1 hr by immunofluorescence assay. Scale bar, 10 μm.

b DNA-binding domain (DBD) of Nur77 is not required for celastrol-induced Nur77 interaction with p62. Interaction of Flag-p62 with Myc-Nur77 or Myc-Nur77-ΔDBD was analyzed in HeLa cells treated with or without celastrol (2 μM) for 1 hr by co-IP assay.

c Nur77-LBD interacts with UBA but not PB1 of p62 in a celastrol-dependent manner. Interaction of 3*Flag-Nur77-LBD with mCherry-p62-PB1 or mCherry-p62-UBA was analyzed in HeLa cells treated with or without celastrol (2 μM) for 1 hr by co-IP assay.

d Nur77-IDR regulates p62 body expansion. Representative images and their quantification illustrate the effect of Nur77 mutants in regulating p62 body formation in *Nur77*$^{-/-}$HeLa cells. *Nur77*$^{-/-}$HeLa cells transfected with mCherry-p62 and the indicated GFP-Nur77 or mutant were treated with or without celastrol (2 μM) and TNFα (20 ng/ml) for 1 hr. The diameter of the big p62 puncta and number of p62 puncta >0.5 μm in each cell was measured. N = 47, 56, 41, 40, 30, 30, 42, 37, respectively. Two-tailed unpaired Student's t test was used for statistical analysis, and data are presented as mean values ± SEM. Scale bar, 10 μm.
