# Supplementary material for: Phase separation of Nur77 mediates celastrol-induced mitophagy by promoting the liquidity of p62/SQSTM1 condensates
Source: Nat Commun. 2021 Oct 13;12:5989. doi: 10.1038/s41467-021-26295-8 (PMC8514450; doi:10.1038/s41467-021-26295-8)
Supplement: Supplementary file 3 — Description of Additional Supplementary Files [file 41467_2021_26295_MOESM3_ESM.docx]

**Description of Additional Supplementary Files**

**Supplementary Movie 1.** Celastrol promotes the growth and fusion of Nur77/p62 bodies. HeLa cells transfected GFP-Nur77 and mCherry-p62 rapidly formed large cytoplasmic bodies through fusion of undetectable micro-sized droplets or detectable droplets after cells were treated with celastrol for 1 hr.

**Supplementary Movie 2.** Celastrol promotes the growth and fusion of Nur77/p62 bodies. HeLa cells transfected GFP-Nur77 and mCherry-p62 rapidly formed large cytoplasmic bodies through fusion of undetectable micro-sized droplets or detectable droplets after cells were treated with celastrol for 1 hr.

**Supplementary Movie 3.** Celastrol promotes the growth and fusion of Nur77/p62 bodies. HeLa cells transfected GFP-Nur77 and mCherry-p62 rapidly formed large cytoplasmic bodies through fusion of undetectable micro-sized droplets or detectable droplets after cells were treated with celastrol for 1 hr.

**Supplementary Movie 4.** Celastrol promotes the nuclear export of Nur77 and the growth of Nur77 bodies in the cytoplasm. GFP-Nur77 transfected into HeLa cells migrated from the nucleus to the cytoplasm when cells were treated with celastrol for 1 hr. Celastrol also promoted the growth and fusion of GFP-Nur77 in the cytoplasm.
