# Supplementary material for: Phase separation of Nur77 mediates celastrol-induced mitophagy by promoting the liquidity of p62/SQSTM1 condensates
Source: Nat Commun. 2021 Oct 13;12:5989. doi: 10.1038/s41467-021-26295-8 (PMC8514450; doi:10.1038/s41467-021-26295-8)
Supplement: Supplementary file 8 — Reporting Summary [file 41467_2021_26295_MOESM8_ESM.pdf]

nature portfolio

Corresponding author(s): Xiao-kun Zhang

Last updated by author(s): Sep 10, 2021

Reporting Summary

Nature Portfolio wishes to improve the reproducibility of the work that we publish. This form provides structure for consistency and transparency in reporting. For further information on Nature Portfolio policies, see our Editorial Policies and the Editorial Policy Checklist.

Statistics

For all statistical analyses, confirm that the following items are present in the figure legend, table legend, main text, or Methods section.

n/a	Confirmed	
☐	☒	The exact sample size (*n*) for each experimental group/condition, given as a discrete number and unit of measurement
☐	☒	A statement on whether measurements were taken from distinct samples or whether the same sample was measured repeatedly
☐	☒	The statistical test(s) used AND whether they are one- or two-sided<br>*Only common tests should be described solely by name; describe more complex techniques in the Methods section.*
☒	☐	A description of all covariates tested
☒	☐	A description of any assumptions or corrections, such as tests of normality and adjustment for multiple comparisons
☐	☒	A full description of the statistical parameters including central tendency (e.g. means) or other basic estimates (e.g. regression coefficient) AND variation (e.g. standard deviation) or associated estimates of uncertainty (e.g. confidence intervals)
☐	☒	For null hypothesis testing, the test statistic (e.g. *F*, *t*, *r*) with confidence intervals, effect sizes, degrees of freedom and *P* value noted<br>*Give P values as exact values whenever suitable.*
☒	☐	For Bayesian analysis, information on the choice of priors and Markov chain Monte Carlo settings
☒	☐	For hierarchical and complex designs, identification of the appropriate level for tests and full reporting of outcomes
☐	☒	Estimates of effect sizes (e.g. Cohen's *d*, Pearson's *r*), indicating how they were calculated

Our web collection on statistics for biologists contains articles on many of the points above.

Software and code

Policy information about availability of computer code

Data collection	The images were taken under a LEICA TCS SP8 confocal laser scanning microscope system (Leica Microsystems GmbH, Mannheim, Germany); FRAP was carried out using Zeiss LSM 880 microscope (Zeiss, Germany); CytoFLEX Flow Cytometry System (Beckman-Coulter, Miami, FL, USA) was used for EGFP-mCherry-COX8 analysis. The size of droplet was determined using a Malvern zetasizer (Malvern instrument, Worcestershire, UK) apparatus. Immunogold-labeled samples were imaged with a Hitachi HT-7800 electron microscope.
Data analysis	GraphPad Prism version 8 (GraphPad software, La Jolla, CA, USA) was used for the preparation of all the graphs and the statistical analyses; FIJI plugin FRAP Profiler was used for measuring the fluorescence intensity at the bleached spot. Image J software version 1.52 (NIH) was used for measuring mitochondrial size, diameter of droplets and quantification to obtain droplets or cells numbers.

For manuscripts utilizing custom algorithms or software that are central to the research but not yet described in published literature, software must be made available to editors and reviewers. We strongly encourage code deposition in a community repository (e.g. GitHub). See the Nature Portfolio guidelines for submitting code & software for further information.

Data

Policy information about availability of data

All manuscripts must include a data availability statement. This statement should provide the following information, where applicable:

- Accession codes, unique identifiers, or web links for publicly available datasets
- A description of any restrictions on data availability
- For clinical datasets or third party data, please ensure that the statement adheres to our policy

Source data are provided with this paper. The source data underlying all Figs. and Supplementary Figs. are provided as the Source Data file. All the other data supporting the findings of this study are available in the article and its supplement information files or from the corresponding author upon reasonable request.

Field-specific reporting

Please select the one below that is the best fit for your research. If you are not sure, read the appropriate sections before making your selection.

- [x] Life sciences
- [ ] Behavioural & social sciences
- [ ] Ecological, evolutionary & environmental sciences

For a reference copy of the document with all sections, see nature.com/documents/nr-reporting-summary-flat.pdf

Life sciences study design

All studies must disclose on these points even when the disclosure is negative.

Sample size	Sample sizes were estimated based on previously published studies [M. J. Hu et al., Molecular Cell 2017 Vol. 66 Issue 1 Pages 141-+; https://doi.org/10.1016/j.molcel.2017.03.008]. For in vivo studies, we found that 4-6 mice are sufficient to show significant differences among groups. Sample size was determined to be adequate based on the magnitude and consistency of measurable differences between groups.
Data exclusions	On principle, data were only excluded for failed experiments, reasons for which included suboptimal activation and microbial contamination.
Replication	All data presented in this study are representative results of at least three independent experiments and all attempts at replication were successful.
Randomization	Samples and animal subjects were allocated randomly.
Blinding	Data acquisition was performed in a blinded fashion and the investigators were blinded to group allocation during data analysis.

Reporting for specific materials, systems and methods

We require information from authors about some types of materials, experimental systems and methods used in many studies. Here, indicate whether each material, system or method listed is relevant to your study. If you are not sure if a list item applies to your research, read the appropriate section before selecting a response.

Materials & experimental systems

n/a	Involved in the study	
☐	☒	Antibodies
☐	☒	Eukaryotic cell lines
☒	☐	Palaeontology and archaeology
☐	☒	Animals and other organisms
☒	☐	Human research participants
☒	☐	Clinical data
☒	☐	Dual use research of concern

Methods

n/a	Involved in the study	
☒	☐	ChIP-seq
☐	☒	Flow cytometry
☒	☐	MRI-based neuroimaging

Antibodies

Antibodies used

For Western blotting, the following antibodies were used:
mouse Anti-Myc (9E10) (Santa Cruz, sc-40, dilution: 1:1000),
mouse anti-FLAG (Sigma-Aldrich, F1804, dilution: 1:1000),
mouse anti-GFP (Santa Cruz, sc-9996, dilution: 1:1000),
rabbit anti-COXII (Abclonal, A3843, dilution: 1:1000),
rabbit anti-LC3 (Abcam, AB51520, dilution: 1:5000),
rabbit anti-Nur77 for HeLa cell (CST, 3960S, dilution: 1:500),
mouse anti-Nur77 for mice (Thermo Fisher Scientific, 14-5965-82, dilution: 1:1000),
mouse anti-SQSTM1/p62 (Abcam, AB56416, dilution: 1:5000),
rabbit anti-Atg7 (HUABIO, SC06-30, dilution: 1:1000),
mouse anti-β-actin (Sigma-Aldrich, A5441, dilution: 1:10000),
mouse anti-GAPDH (Proteintech, 60004, dilution: 1:10000),
Goat Anti-Mouse IgG F(ab')2 Secondary Antibody, HRP conjugate (1:10000, Pierce Chemical 31436),
Goat Anti-Rabbit IgG F(ab')2 Secondary Antibody, HRP conjugate (1:10000, Pierce Chemical 31461),
Peroxidase-conjugated AffiniPure Goat Anti-Mouse IgG (H+L) Secondary Antibody (1:10000, Jacksonimmuno 115-035-003),
Peroxidase-conjugated AffiniPure Goat Anti-Rabbit IgG (H+L) Secondary Antibody (1:10000, Jacksonimmuno 115-035-003)

For immunoprecipitation, mouse anti-FLAG (Sigma-Aldrich, F1804, dilution: 1:100) was used.

For immunofluorescence staining, the following antibodies were used:

rabbit anti-Nur77 for HeLa cell (CST, 3960S, dilution: 1:100),
rabbit anti-Nur77 (M-210) (Santa Cruz, sc-5569, dilution: 1:50),
rabbit anti-Nur77 for mice (Affinity, DF7850, dilution:1:200),
rabbit anti-CD68 (Abcam, AB955, dilution: 1:200),
rabbit anti-SQSTM1/p62 (Abcam, AB56416, dilution: 1:400),
mouse anti-Myc (9E10) (Santa Cruz, sc-40, dilution: 1:100),
mouse anti-FLAG (Sigma-Aldrich, F1804, dilution: 1:400),
mouse anti-HA (Santa Cruz, sc-7392, dilution: 1:100),
rabbit anti-LC3 (MBL, PM036, dilution: 1:200),
mouse anti-Hsp60 (Santa Cruz, sc-13115, dilution: 1:100),
rabbit anti-FIP200 (CST, 12436, dilution: 1:100),
rabbit anti-ATG16L1 (Proteintech, 19812, dilution: 1:100),
rabbit anti-WIPI2 (Proteintech, 15432, dilution: 1:100),
mouse anti-ULK1 (Santa Cruz, Sc-390904, dilution: 1:50),
Mito Tracker Red FM (Thermo Fisher Scientific, M22425, dilution: 1:20000),
Mito Tracker Deep Red FM (Thermo Fisher Scientific, M22426, dilution: 1:20000),
Lyso Tracker Deep Red FM (Thermo Fisher Scientific, L12492, dilution: 1:20000),
Goat anti-Rabbit IgG (H+L) Cross-Adsorbed Secondary Antibody, Cy5 (Thermo Fisher Scientific, A10523, dilution: 1:200),
Goat anti-Mouse IgG (H+L) Cross-Adsorbed Secondary Antibody, Cy5 (Thermo Fisher Scientific, A10524, dilution: 1:200),
Cy3-AffiniPure Goat Anti-Rabbit IgG (H+L) (Jackson, 111-165-003, dilution: 1:200),
Cy3-AffiniPure Goat Anti-Mouse IgG (H+L) (Jackson, 115-165-003, dilution: 1:200),
FITC-AffiniPure Rabbit Anti-Goat IgG (H+L) FITC (Yeasen, 33707ES60, dilution: 1:200).

For Immunogold electron microscopy, the following antibodies were used:
rabbit anti-Nur77 (CST, 3960S, dilution: 1:100),
mouse anti-SQSTM1/p62 (Abcam, AB56416, dilution: 1:100),
rabbit anti-LC3 (MBL, PM036, dilution: 1:200),
Goat Anti-rabbit IgG/Gold (AB-0295G-Gold, 15 nm), Goat Anti-mouse IgG/Gold (AB-0296R-Gold, 10 nm), (Leading Biology Inc. (California, USA), AB-0295G-Gold, dilution: 1:100).

Validation

1. Myc (9E10): mouse, rat, human, monkey, feline and canine. WB, IP, IF, IHC(P), FCM and ELISA. (https://www.scbt.com/p/c-myc-antibody-9e10?requestFrom=search)
2. FLAG: mammalian, plant, and bacterial. WB, IP , IHC, IF and ICC. (https://www.sigmaaldrich.cn/CN/en/product/sigma/f1804?context=product)
3. GFP: WB, IP, IF, IHC(P), FCM and ELISA. (https://www.scbt.com/p/gfp-antibody-c-2?productCanUrl=gfp-antibody-c-2&_requestid=697600)
4. COXII: human. WB, IHC. (https://abclonal.com.cn/catalog/A3843)
5. LC3 (Abcam AB51520): Human. Flow Cyt, IHC-Fr, WB, ICC/IF. (https://www.abcam.cn/lc3b-antibody-ab51520.html)
6. β-actin: pig, Hirudo medicinalis, bovine, rat, canine, feline, human, rabbit, carp, mouse, guinea pig, chicken, sheep. WB, IHC (p), IF, ELISA. (https://www.sigmaaldrich.cn/CN/en/product/sigma/a5441?context=product)
7. Nur77 (CST 3960S): human. Flow Cyt, IHC, WB, ICC/IF. (https://www.cellsignal.cn/products/primary-antibodies/nur77-d63c5-xp-rabbit-mab/3960?_=1631173079519&Ntt=3960s&tahead=true)
8. Nur77 (Thermo Fisher Scientific 14-5965-82): mouse. WB. (https://www.thermofisher.cn/cn/zh/antibody/product/Nur77-Antibody-clone-12-14-Monoclonal/14-5965-82)
9. SQSTM1/p62 (Abcam AB56416): human, mouse. IHC-P, IP, WB, ICC/IF, Flow Cyt. (https://www.abcam.cn/sqstm1--p62-antibody-2c11-bsa-and-azide-free-ab56416.html)
10. Atg7: human, mose. WB, ICC/IF, IHC-P, IP, FC. (http://www.huabio.cn/product/Apg7-antibody-ET1610-53)
11. GAPDH: human, mouse, rat, yeast, plant. ChIP, CoIP, FC, IF, IHC, IP, WB (https://www.ptgcn.com/Products/GAPDH-Antibody-60004-1-Ig.htm)
12. Nur77 (M-210) (Santa Cruz sc-5569): human, WB, ELISA, IHC. a discontinued antibody.
13. Nur77 (Affinity DF7850): Human, Mouse, Rat. WB,IHC,IF/ICC,ELISA(peptide). (http://www.affbiotech.com/goods-11554-DF7850-NUR77+Antibody.html)
14. FIP200: human, mouse. WB, IP. (https://www.cellsignal.cn/products/primary-antibodies/fip200-d10d11-rabbit-mab/12436?site-search-type=Products&N=4294956287&Ntt=12436&fromPage=plp&_requestid=746916)
15. ATG16L1: human, mouse. IP, WB, ELISA. (http://www.ptgcn.com/products/ATG16L1-Antibody-19812-1-AP.htm)
16. WIPI2: human, mouse. ELISA, IF. (http://www.ptgcn.com/products/WIPI2-Antibody-15432-1-AP.htm)
17. Hsp60:mouse, rat, human and origin. WB, IP, IF, IHC(P) and ELISA. (https://www.scbt.com/p/hsp-60-antibody-h-1?productCanUrl=hsp-60-antibody-h-1&_requestid=723151)
18. ULK1: mouse, rat, human. WB, IHC(P), ELISA (https://www.scbt.com/p/ulk1-antibody-f-4?requestFrom=search)
19. LC3 (MBL, PM036): Human, Mouse, Rat, Hamster, Zebrafish. FCM, ICC, IHC, IP, WB. (https://www.mblbio.com/bio/g/dtl/A/index.html?pcd=PM036)
20. HA: WB, IP, IF, FCM , ELISA. (https://www.scbt.com/p/ha-probe-antibody-f-7?requestFrom=search)
21. CD68: human, mouse. WB, ICC, IHC-P.(https://www.abcam.cn/cd68-antibody-kp1-ab955.html)

Eukaryotic cell lines

Policy information about cell lines

Cell line source(s)

HeLa and HEK293T were purchased from the American Type Culture Collection (Manassas, VA, USA).Primary mouse

	embryonic fibroblasts (MEFs) and p62-/- MEFs were obtained from M.T.D.-M and J.M.'s lab.
Authentication	The cell lines have been validated by the suppliers. The growth of the cell lines used were verified with the supplier's data sheets with stable morphology feature and pharmacological response. HeLa/control and Nur77-/- satble cells were generated using CRISPER/Cas9 techonlogy and validated by STR typing (Genetic testing biotechnology, Suzhou, China).
Mycoplasma contamination	Cell lines were tested negative for mycoplasma.
Commonly misidentified lines (See ICLAC register)	No cell lines used are listed in the database of commonly misidentified cell lines.

Animals and other organisms

Policy information about studies involving animals; ARRIVE guidelines recommended for reporting animal research

Laboratory animals	Wild-type (C57BL/6J, Stock No.: 000664) and Nur77-/-mice (Nur77 C57BL/6J, Stock No.: 006187) were purchased from the Jackson Laboratory (Bar Harbor, Maine, USA). All experiments were performed on female cohorts at 2 months of age (young mice) and 2 years of age (old mice)(8 weeks old and 2 years old). All mice were maintained under conditions of controlled temperature housed in pathogen-free facilities, in a 12 hr dark/light cycle with temperatures of 22.5 °C and 50%-55% humidity at the Laboratory Animal Center in Xiamen University.
Wild animals	No wild animals were used in this study.
Field-collected samples	No field-collected samples were used in this study.
Ethics oversight	The protocols for animal studies were approved by the Animal Care and Use Committee of Xiamen University, and all mice were handled in accordance with the ''Guide for the Care and Use of Laboratory Animals'' and the ''Principles for the Utilization and Care of Vertebrate Animals".

Note that full information on the approval of the study protocol must also be provided in the manuscript.

Flow Cytometry

Plots

Confirm that:

- [x] The axis labels state the marker and fluorochrome used (e.g. CD4-FITC).
- [x] The axis scales are clearly visible. Include numbers along axes only for bottom left plot of group (a 'group' is an analysis of identical markers).
- [x] All plots are contour plots with outliers or pseudocolor plots.
- [x] A numerical value for number of cells or percentage (with statistics) is provided.

Methodology

Sample preparation	Nur77-/-HeLa cells were cotransfected with Nur77 or mutants together with EGFP-mCherry-COX8, and then treated with celastrol and TNFα.
Instrument	Beckman-Coulter, Miami, FL, USA
Software	CytoFLEX Flow 731 Cytometry System
Cell population abundance	Flow cytometry scatter used to detect the mCherry-COX8 exposure cells.
Gating strategy	Single and living cells were gated by P1. The events with very low FSC and SSC, as well as those with high FSC and SSC were eliminated. These events represent debris, cell fragments and doublets. HeLa cells without transfection of EGFP-mCherry-COX8 were used as blank control to set the FITC-positive gate. HeLa cells transfected with EGFP-mCherry-COX8 were selected by FITC gate. Measurements EGFP-mCherry-COX8 were made using dual-excitation pH measurements at FITC (488nm, pH 7) and PE (561 nm, pH 4) lasers, respectively. For each sample, 10,000 events of FITC positive cells were collected and subsequently gated for mCherry-COX8 exposure cells but no EGFP-mCherry-COX8 double-positive cells. (Fig.S1A).

- [x] Tick this box to confirm that a figure exemplifying the gating strategy is provided in the Supplementary Information.
